# MicroRNA and Other Non-Coding RNAs in Epstein–Barr Virus-Associated Cancers

**DOI:** 10.3390/cancers13153909

**Published:** 2021-08-03

**Authors:** Kin Israel Notarte, Suranga Senanayake, Imee Macaranas, Pia Marie Albano, Lucia Mundo, Eanna Fennell, Lorenzo Leoncini, Paul Murray

**Affiliations:** 1 Faculty of Medicine & Surgery, University of Santo Tomas, Manila 1008, Philippines or krnotarte@ust.edu.ph (K.I.N.); imcrns@gmail.com (I.M.); 2 Mater Misericordiae University Hospital, D07AX57 Dublin, Ireland; slsamara@physicians.ie; 3 Research Center for the Natural and Applied Sciences, Molecular Diagnostics Research Group, University of Santo Tomas, Manila 1015, Philippines; psalbano@ust.edu.ph; 4 Department of Medical Biotechnologies, Section of Pathology, University of Siena, 53100 Siena, Italy; lucia.mundo.ml@gmail.com (L.M.); Lorenzo.leoncini@dbm.unisi.it (L.L.); 5 Health Research Institute, University of Limerick, V94 T9PX Limerick, Ireland; Eanna.Fennell@ul.ie; 6 Institute of Immunology and Immunotherapy, University of Birmingham, Birmingham B15 2TT, UK; 7 Institute of Molecular and Translational Medicine, Palacký University in Olomouc, Hněvotínská 5, 779 00 Olomouc, Czech Republic

**Keywords:** microRNAome, miRNA, EBV, immune evasion, carcinogenesis, classical Hodgkin’s lymphoma, Burkitt lymphoma, diffuse large B-cell lymphoma, nasopharyngeal carcinoma, gastric carcinoma

## Abstract

**Simple Summary:**

Epstein–Barr virus (EBV) is associated with a variety of malignancies. In this review, we discuss EBV-encoded microRNAs and ncRNAs and consider how their detection could aid in the diagnosis, prognostication, and monitoring of treatment in patients with EBV-associated malignancies, including classical Hodgkin’s lymphoma (cHL), Burkitt lymphoma (BL), diffuse large B-cell lymphoma (DLBCL), nasopharyngeal carcinoma (NPC), and gastric carcinoma (GC).

**Abstract:**

EBV is a direct causative agent in around 1.5% of all cancers. The oncogenic properties of EBV are related to its ability to activate processes needed for cellular proliferation, survival, migration, and immune evasion. The EBV latency program is required for the immortalization of infected B cells and involves the expression of non-coding RNAs (ncRNAs), including viral microRNAs. These ncRNAs have different functions that contribute to virus persistence in the asymptomatic host and to the development of EBV-associated cancers. In this review, we discuss the function and potential clinical utility of EBV microRNAs and other ncRNAs in EBV-associated malignancies. This review is not intended to be comprehensive, but rather to provide examples of the importance of ncRNAs.

## 1. Introduction

In 2018, an estimated 2.2 million infection-attributable cancer cases were diagnosed worldwide. A conservative estimate suggests that almost 1.4 million of these were associated with oncogenic viruses [1], including the hepatitis B virus, hepatitis C virus, Kaposi sarcoma-associated herpesvirus, human T lymphotropic virus type 1, human papillomaviruses, and Epstein–Barr virus (EBV) [2]. The oncogenic properties of these viruses are directly related to their ability to activate processes needed for cellular proliferation, survival, migration, and immune evasion [3]. Among these viruses, EBV, formerly designated as the human herpesvirus type 4 (HHV-4), is a *y*-herpesvirus containing a linear, double-stranded DNA genome of ~172 kilobase pairs (kbp), encoding nearly 80 proteins and 46 functional small untranslated RNAs [2,4]. The genetic material of EBV is enclosed in an icosahedral nucleocapsid surrounded by the viral tegument and lipid-containing outer envelope. EBV is transmitted through oral contact, particularly in the early years of life, usually without causing disease [5]. EBV can also be transmitted through organ transplantation and blood transfusion [6]. The life cycle of EBV primarily involves the infection of lymphocytes and potentially epithelial cells. Although EBV often exists as an asymptomatic infection, it is involved in the development of about 1.5% of all cancers worldwide [7]. In fact, EBV was the first virus to have been directly associated with cancer in humans. EBV-associated neoplasms affect both immune-competent and immunocompromised hosts, including, for example, some organ transplant recipients. Immune dysregulation and genetic susceptibility are probable co-factors in most, if not all, EBV-associated cancers [8].

The EBV life cycle begins when the virion enters naïve B-lymphocytes, or in some cases, perhaps memory B-lymphocytes, probably following initial infection of epithelial cells [9]. Although the exact mechanisms of EBV entry into epithelial cells are becoming clearer, how the virus crosses the epithelial barrier to infect B cells in vivo remains unknown [10]. The virus may infect the epithelial cells, replicate, and then be released to infect B cells in the underlying areas, but there is no direct evidence for this and normal epithelial cells appear to be resistant to infection from the apical (i.e., mucosal) side [11]. If the virus is unable to infect epithelial cells directly, then it may be able to traverse the epithelial membrane barrier to access B cells, perhaps when the epithelial lining is damaged, or becomes leaky during inflammation [12]. Conversely, on exit to the oropharynx, there is some evidence that transfer infection of a virus from B cells to epithelial cells can occur via the basolateral surface [12].

Following cell entry, the viral genome is released into the nucleus where it becomes circularized, an event that maintains the EBV genome as an extrachromosomal episome that is readily replicated and is used as a marker of viral clonality [13]. Furthermore, the EBV genome carrying few epigenetic tags associates itself with histones and becomes methylated due to the similarity of its nucleosomal structure with that of the host genome. DNA methylation and histone modification are vital epigenetic mechanisms that regulate gene expression necessary for completing the viral life cycle [14]. To ensure its persistence in infected B cells, EBV enters the latency phase resulting in the silencing of some viral genes, an event that is crucial for evading host cell immunity [9]. There are different latency states. For instance, expression of the latency III or the ‘growth program’ consisting of six EBV nuclear antigens (EBNA-1, -2, -3A, -3B, -3C, -LP) and three latent membrane proteins (LMP-1, -2A, -2B) results in the proliferation and immortalization of primary B cells [15]. Latency II or the ‘default program’ consisting of EBNA1, LMP1, LMP2A, and LMP2B, is expressed in EBV-infected germinal center B cells. The latency I program, which is limited to the expression of only one protein, EBNA1, is responsible for maintaining viral episomes in dividing memory B cells [13]. Latency 0, characterized by the absence of viral gene expression, is observed in non-dividing memory B cells [9]. During these different latency programs, the virus utilizes non-coding RNAs, including viral microRNAs [16]. Latency is halted and viral reactivation begins when memory B cells terminally differentiate to plasma cells. The new virions are then released from B cells and may infect epithelial cells where the virus is amplified for cell-to-cell spread or infection of a new host [17]. Compared to B cells, the nature of EBV infection of epithelial cells is less well understood [18].

The small non-coding, non-polyadenylated RNAs EBER-1 and EBER-2 are also abundantly expressed in both EBV-infected non-malignant and cancerous cells [19]. Owing to the significantly longer half-life of EBER-1, it is usually present at ten-fold higher levels compared with EBER-2 [20]. The EBERs do not code any proteins and their abundance makes them a valuable diagnostic tool for EBV detection; using in situ hybridization, the detection of EBER has been established as the most sensitive and practical method for detecting EBV [21]. However, the precise contribution of the EBERs to the viral life cycle and to malignant transformation remain unclear. The EBERs are not required for EBV-induced transformation of primary B-lymphocytes, but assemble into stable ribonucleoprotein particles with the La and L22 proteins [22,23], and bind the interferon-inducible, double-stranded RNA-activated protein kinase PKR, suggesting that they may be involved in suppressing the antiviral effects of the interferons [24]. EBER2 RNA may regulate the levels of LMP2 and it has been shown to exist in a complex with PAX5; this complex can regulate LMP2A/B and LMP1 expression [25]. Knockdown of EBER2 also decreased EBV lytic replication [26]. In a recent study, EBER2 was shown to be able to substitute for the Marek’s disease virus telomerase RNA-like viral RNA [27].

Apart from its non-coding EBER, EBV can also express a number of microRNAs (miRNAs). miRNAs are highly conserved, small, non-coding RNAs important for gene expression in various organisms, including humans. Although small, miRNAs outnumber coding sequences in the human genome. Their role in gene expression is not limited to normal functions but are also important in the development of disease. Of particular interest, here is the dysregulation of miRNAs in cancer [28,29,30,31]. miRNA genes are transcribed into primary RNA (pri-miRNA) by RNA polymerase II or III; and then cleaved into precursor miRNA (pre-miRNA) by a microprocessor complex comprised of endonuclease enzymes, DROSHA or DGCR8. The pre-miRNA is then transported from the nucleus into the cytoplasm via a nucleocytoplasmic exporter, which contains exportin-5 (XPO5) and RAN-GTP. In the cytoplasm, the pre-miRNA is cleaved into a miRNA duplex by a complex of DICER and transactivating response RNA-binding protein (TRBP), and further cleavage results in the generation of the mature miRNA. The mature miRNA is incorporated within the RNA induced silencing complex (RISC) and Argonaute proteins (Ago2). This protein complex is responsible for regulating translation of the target mRNAs [29,30]. Dysregulation in any step of miRNA biogenesis, for example by genetic, epigenetic, and transcriptional mechanisms, may result in alterations in mRNA translation. For instance, upregulation of oncogenic miRNAs (oncomiR) (for example, those involved in regulation of the cell cycle), and/or downregulation of tumor suppressive miRNAs (tumor-suppressor miR) can contribute to carcinogenesis [30,32]. Some well-studied oncomiRs include miR-155, which is over-expressed in some types of lymphoma and leukemia [33,34]. Let-7 miRNA is an example of a tumor-suppressor miR, and is downregulated in lung cancer [35]. However, some miRNAs can be oncogenic or tumor suppressive depending upon the type of cancer. For example, miR-17-92 is upregulated in lung cancer [36], but downregulated in breast cancer [37]. Moreover, a single miRNA may be involved in more than one biological pathway [28,29,30,31,32].

Apart from miRNAs, other non-coding RNAs including long non-coding RNAs (lncRNAs) are also increasingly linked to cancer. The first lncRNA shown to be involved in cancer was HOX Antisense Intergenic RNA (HOTAIR), which is upregulated in breast cancer [38]. Another lncRNA, the Metastasis-Associated Lung Adenocarcinoma Transcript 1 (MALAT1), is also upregulated in lung and colorectal cancers [39,40].

Due to the observed changes in the expression of miRNAs in cancer, miRNAs in circulating body fluids are being investigated as potential minimally invasive biomarkers that could help in both the diagnosis and monitoring of patients [32,41,42]. In this review, we consider the functions of the EBV-encoded miRNAs and of other ncRNAs, in normal and cancer cells, and highlight their potential clinical utility.

## 2. Classical Hodgkin’s Lymphoma

Classic Hodgkin’s lymphoma (cHL) is the prototypic ‘inflamed lymphoma’, having an unusual histology characterized by usually single malignant Hodgkin’s–Reed–Sternberg (HRS) cells surrounded by a prominent inflammatory infiltrate [43,44]. HRS cells secrete chemokines and other soluble factors that recruit and retain the immune cells to create a tissue microenvironment that functions to support the survival and growth of the HRS cells, while at the same time allowing them to evade tumor-specific immunity [45].

Constitutive activation of a family of transcription factors, collectively referred to as the nuclear factor kappa B (NF-κB) family, is a key pathogenic feature of HRS cells [46]. NF-κB signaling contributes essential functions in HRS cells, which could be especially important in the absence of a functional BCR pathway, which is a hallmark of HRS cells. For example, it has been shown that inhibition of this pathway leads to increased sensitivity of HL cell lines to apoptosis after growth factor withdrawal and impaired tumorigenicity in severe combined immunodeficiency (SCID) mice [47,48].

EBV-positive HRS cells display a strict latency II type, in which there is consistent expression of EBNA-1, LMP-1, and LMP-2A [49,50,51,52]. While the contribution of these EBV latent proteins to the pathogenesis of cHL is increasingly better-understood [53], the roles of the EBV miRNAs have only just begun to be explored.

**Figure 1:**
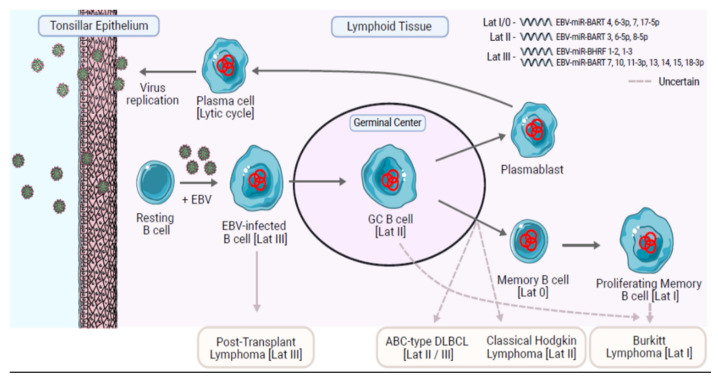
A model to explain the life cycle of EBV in B cells and the origin of EBV-positive B cell lymphomas. Trafficking B cells are presumed to become infected as they pass close to epithelium, potentially in the tonsils. The infected B cells probably then undergo proliferation driven by the Latency III virus gene expression program. The infected B cells enter a germinal center reaction in which only the EBNA1, LMP1, and LMP2A viral proteins are expressed (Latency II). LMP1 is a CD40 homologue and LMP2A is a B cell receptor (BCR)-mimic. Together LMP1 and LMP2A are thought to provide the signals necessary for the EBV-infected cells to survive a germinal center reaction and to differentiate into memory B cells and plasma cells. Plasma cells replicate the virus leading to the release of new virions, which can pass out into the oropharynx for infection of other susceptible hosts. Memory B cells provide the vehicle for long-term virus persistence and are characterized by an absence of virus protein expression (Latency 0). Memory B cells may switch on EBNA1 expression when they proliferate (Latency I). Distinct patterns of miRNA expression in ‘normal’ EBV-infected B cells are associated with Latency III and the other restricted forms of latency II/I/0 (listed in top right of figure). Latency I/0 is characterized by a strong expression of EBV BART4, 6-3p, 7 and a significantly higher expression of miR-17-5p compared to latency II showing modest level of EBV BART3*, 6-5p, and 8-5p. Latency III is characterized by intermediate level of EBV BART7*, 10, 11-3p, 13*, 14*, 15, 18-3p, and EBV-miR-BHRF1-2, 1-3, not expressed in the other forms of latency. The origin of the EBV-associated B cell lymphomas remains uncertain. Assumptions of origin are based largely on resemblance to normal B cell phenotypes and virus latency programs. BL tumors phenotypically resemble normal GC B cells and are probably derived from this stage of B cell differentiation. However, because BL usually expresses a Lat I phenotype, it has been suggested by others that BL could be derived from proliferating memory B cells. Figure made with Servier Medical Art. 2021. SMART—Servier Medical ART (online). Available online: https://smart.servier.com/, accessed on 5 July 2021.

Before considering the contribution of EBV miRNA to the pathogenesis of B cell lymphomas, including cHL, it is important to first reprise the most likely mechanism used by EBV to persist in the lymphoid compartment. In this model, originally proposed by Thorley-Lawson, EBV infection of naïve B cells drives them into a proliferative state in which the full latency III program of virus gene expression occurs [54,55] (Figure 1). Upon further differentiation, the EBV-infected B cells enter a germinal center (GC) reaction, where they express a latency II program. Eventually, the EBV-infected memory B cells that emerge from the germinal center shut down virus gene expression (latency 0), only occasionally switching on EBNA1 expression when they are required to proliferate (latency I) [56,57].

Profiling of EBV miRNAs at these different stages of EBV infection has shown that the transit from EBV-driven growth, characterized by the latency III program of lymphoblastoid cell lines, into more restricted forms of latency in EBV-infected germinal center and memory B cells is also associated with changes in the expression of the EBV miRNA [58]. EBV-immortalized lymphoblastoid cells express a subset of BART miRNA (including approximately half of Cluster 2), as well as three of the four BHRF1 miRNAs, which are subsequently turned off in germinal center B cells and memory B cells [58]. EBV-infected germinal center B cells and memory B cells upregulate the remaining BART miRNAs by 5–10-fold [58].

Notable differences in the levels of the different BART and BHRF1 miRNA are observed in different EBV-positive tumors, even of the same type [59,60,61]. It has been suggested that this could be because of strain variations [62,63]; in one study, variations in transformation potential between different virus isolates were associated with different levels of BHRF1 miRNA [64]. There could also be technical reasons for the differences observed between studies. These technical variations are potentially exacerbated in cHL in which the HRS cells are often scarce and the sensitivity of miRNA detection is compromised by the large numbers of non-malignant cells included in a bulk analysis of the tumor.

**Figure 2:**
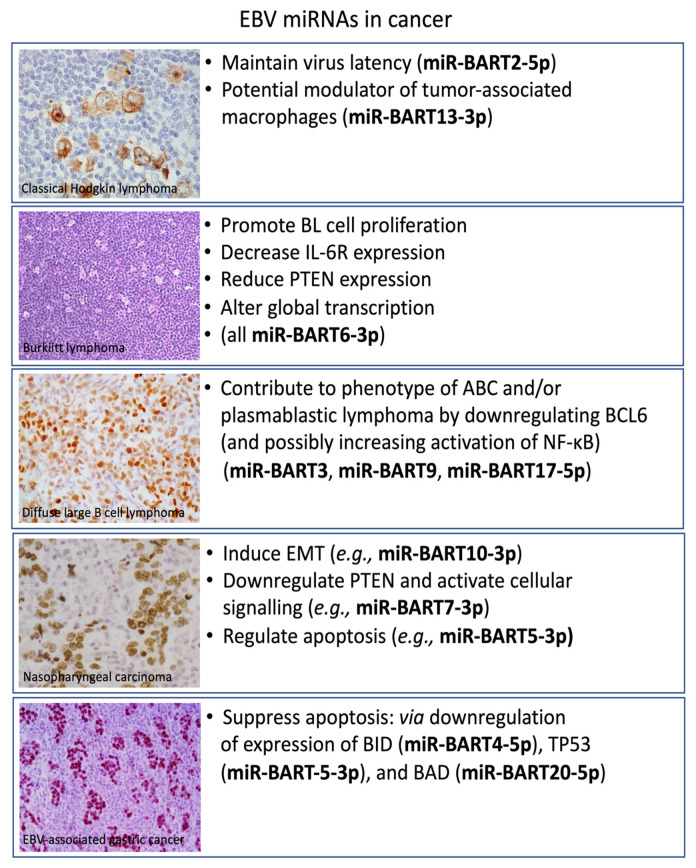
Functions of selected EBV miRNAs in the pathogenesis of the different EBV-associated malignancies. Depicted are EBV-positive classical Hodgkin’s lymphoma showing EBV-LMP1 expression in HRS cells. Burkitt lymphoma showing the typical starry sky appearance on H&E. EBNA2 expression is shown in a Lat III-expressing diffuse large B cell lymphoma. EBNA1 expression is depicted in the two major EBV-associated epithelial neoplasms, nasopharyngeal carcinoma, and gastric carcinoma.

Notwithstanding these caveats, a limited number of studies have suggested potential roles for EBV miRNA in the pathogenesis of cHL. Thus, BART2-5p has been shown to be expressed in cHL; it can act as an antisense miRNA to the EBV DNA polymerase BALF5 [65]. As a result, the level of BALF5 protein and in turn the production of infectious virions is decreased; this is of potential importance in the pathogenesis of EBV-positive cHL, in which entry to the lytic cycle appears to be downregulated and undetectable in most cases [66]. BART2-5p has also been shown to reduce B cell receptor signaling, which might be important in maintaining latency in cHL, in which there is a characteristic loss of BCR functions [67] (Figure 2). Other EBV genes, including *LMP1,* can also reduce the expression of BCR signaling components in B cells [68].

It has been known for some time that EBV-miRNAs can be transferred to recipient cells via exosomes [69]. BART13-3p is one of the most highly expressed viral miRNA in cHL, and can be released into the circulation via exosomes [61]. BARTs present in exosomes derived from EBV-positive cells have been shown to induce changes in the phenotype of macrophages, which include increased production of cytokines, such as the pro-inflammatory cytokine, tumor necrosis factor (TNF)-α, and the immunosuppressive cytokine, IL-10 [70]. Thus, in this way, BART can shift macrophage phenotypes towards a pro-tumor state that can reduce host responses to EBV. It is noteworthy that infiltration by immunosuppressive macrophages is a poor prognostic indicator in cHL [71].

EBV also influences host miRNA expression in cHL. For example, Navarro et al. observed a subset of 10 host miRNAs the expression of which was influenced by the presence of EBV [72]. Among these, miR-96, -128a, and -128b were selectively downregulated in EBV-positive cHL. The authors also reported a distinctive signature of 25 miRNAs that were differentially expressed between cHL and reactive lymph nodes [72]. Among the differentially expressed miRNAs, miR-21, miR-30e/d, miR-92b, and miR-124a were reported to be highly upregulated in HL, and were described as prognostic biomarkers [73,74] (Table 1).

Limited data are available regarding the expression of lncRNA in cHL (Table 1). Tayari et al. defined a lncRNA profile in HL cell lines, which showed a pattern different to that seen in naïve and memory B cells [75,76], but more similar to that observed in GC B cells. Three lncRNAs (FLJ42351, LINC00116, and LINC00461) showed a tumor cell-specific expression in cHL cell lines and in cHL tissues. However, the majority of ncRNAs have not yet been thoroughly investigated and little is known about their function and role in cHL pathogenesis. Fan et al. observed that NEAT1 might contribute to HL progression by promoting cell proliferation and invasion capability via miR-448 mediated doublecortin like kinase 1 (DCLK1) expression [77]. Furthermore, Liang et al. identified a set of 18 lncRNAs (GGTA1P, PCBP1-AS1, GK3P, IL10RB-AS1, PGM5-AS1, HCG18, CHRM3-AS2, PSMD6-AS2, SNHG6, LOC102606465, LOC100190986, GAS5, MIR29B2, PRKCQ-AS1, ITGB2-AS1, MIR142, LOC101060091, and LINC00926) whose deregulated expression was observed in late-relapse HL samples compared to early-relapse HL [78]. Another study demonstrated that the overexpressed lncRNA H19 was positively correlated with the proliferation of HL cells via the AKT pathway and negatively associated with overall survival (OS) of HL patients [79].

**Table 1:** Clinical potential of miRNAs/lncRNAs in cHL.

miRNA/ncRNA	Putative Role	Clinical Potential	References
**25-miRNA**	OncomiR	Diagnostic biomarker	[72]
**has-miR-21**	OncomiR	Prognostic biomarker	[73]
**has-miR-30e/d**	OncomiR	Prognostic biomarker	[73]
**has-miR-92b**	OncomiR	Prognostic biomarker	[73]
**has-miR-124a**	OncomiR	Prognostic biomarker	[74]
***18-lncRNA***	Oncogene	Prognostic biomarker	[78]
***FLJ42351***	Oncogene	Diagnostic biomarker	[75]
***LINC00116***	Oncogene	Diagnostic biomarker	[76]
***LINC00461***	Oncogene	Diagnostic biomarker	[76]
***lncRNA H19***	Oncogene	Prognostic biomarker	[79]

## 3. Burkitt Lymphoma

Burkitt lymphoma (BL) is a highly aggressive B cell non-Hodgkin’s lymphoma (NHL), which manifests in three distinct variants: endemic (eBL), sporadic (sBL), and immunodeficiency-associated (idBL). Each variant exhibits differences in terms of epidemiology, geographical distribution, clinical presentation, genetic features, and association with EBV [80,81].

eBL is most common in equatorial Africa, while sBL is found at a lower incidence throughout the world. Both sBL and eBL have a predilection for extranodal sites (e.g., jaw, kidney, distal ileum/proximal cecum, central nervous system) in comparison to idBL, which often presents with bulky lymph node involvement and occurs commonly in patients infected with the human immunodeficiency virus (HIV) [82]. The hallmark of BL is the constitutive activation of the *MYC* oncogene driven by its juxtaposition to one of the immunoglobulin genes. In 80% of cases, the translocation is between the telomeric region of chromosome 8 and the immunoglobulin heavy chain gene (*IgH*) on chromosome 14 [t(8:14)] [80,81,82].

*MYC* regulates BL cell fate in a direct mode at the transcriptional level and indirectly at the translational level by influencing the miRNA profile [83,84,85,86,87,88,89]. Indeed, the three subtypes of BL share a homogenous cellular miRNA profile, with only marginal miRNA expression differences, while revealing a strong dysregulation of several *MYC*-regulated miRNAs when compared to normal germinal center B cells [83,84,85,86,87,88,89]. This suggests a characteristic *MYC*-induced miRNA expression profile wherein *MYC* is able to reduce as well as to increase the expression of miRNAs involved in B-cell malignancies. For example, miR-17-92 cluster gene, reported to be activated by *MYC*, encodes for six distinct miRNAs (miR-17, miR-18a, miR-19a, miR-19b, miR-20a, miR-92) that suppress chromatin regulatory genes and the apoptosis regulator *BIM,* acting together with *MYC* to accelerate tumor development [90]. Multivariate analysis describes upregulated miR-17 as a significant predictor of shortened OS [90]. *MYC* is also able to induce the expression of miR-9* [91,92]. Remarkably, downregulation of miR-9*, as well as miR-34b, has been described as a diagnostic tool which can define a subset of BL cases in which the *MYC* translocation cannot be detected [92]. Several other MYC-regulated miRNAs implicated in B cell lymphoma are dysregulated in BL [84]. Among the most studied is miR-let-7 the downregulation of which contributes to maintain MYC-induced growth in BL cell lines [93]. miR-21 and miR-155 promote the progression of BL by activating PI3K/AKT signaling [94].

The three BL subtypes differ with respect to their EBV association; the virus is detectable in the neoplastic cells of almost all patients affected by eBL, in approximately 15–30% of cases of sBL, and in 25–40% of idBL [95,96,97]. While EBNA-1 and the EBERs were assumed to be the only EBV genes expressed in eBL, later studies revealed that a small proportion of EBV-positive BL has a novel form of latency with a broader gene expression profile. In this so-called Wp-restricted form of BL, the *BHRF1* gene is also expressed [98,99,100,101].

EBNA-1 and the EBERs are implicated in preventing apoptosis and enhancing tumor cell survival [102,103]. For example, EBNA1 regulates cell viability through NOX2 [87]. EBNA-1 is able to induce the overexpression of hsa-miR-127, which results in the impairment of B-cell differentiation by modulating the expression of BLIMP-1 and XBP-1, two key regulators of terminal B cell differentiation [104]. The EBERs might also contribute to the malignant phenotype of BL; they are implicated in resistance to apoptosis in BL cells and can increase expression of IL-10, an autocrine growth factor for BL cells [105].

Viral miRNAs represent an alternative mechanism adopted by EBV to contribute to BL pathogenesis. Some EBV miRNAs are highly expressed in BL; approximately 2.7% of the miRNAs detected in eBL samples are EBV-miRNAs. BART7, -10, -11-3p, -6-3p, and -17-5p are the most highly expressed BART miRNAs [106,107]. BHRF1 miRNAs are nearly restricted to latency III and thus are hardly detectable in BL samples [108].

EBV miRNAs play important roles in maintaining viral latent infection and in modulating major cell changes without eliciting a strong adaptive immune response. It seems that BL cells depend in part upon BART miRNAs to exhibit their tumor-related properties [109]. EBV BART6-3p is important for maintaining the viral latent phase and has been shown to work synergistically with the cellular miR-142 and miR-197 to promote the proliferation of BL cells via the targeting of PTEN and IL-6 signaling [110,111]. Furthermore, EBV BART6-3p behaves as an oncomiR interfering with the function of important cell signaling pathways, including NF-κB and Akt/PI3K, thus affecting the global gene expression of EBV-associated BL [112,113,114].

It is important to note that while eBL expresses varying levels of EBV miRNAs, to date no association between the expression of EBV-encoded miRNAs and patient outcome, or other clinical features, has been reported [107]. On the contrary, high expression of some host miRNA such as miR-17, miR-21, and miR-23a are associated with poorer outcome in pediatric BL [90,115], and are correlated with clinicopathologic parameters (i.e., tumor staging, size > 6 cm) suggesting they may be candidates for monitoring therapeutic efficacy [107]. The expression of miR-10a-5p is significantly lower in jaw tumors relative to abdominal tumors, and in eBL patients who did not survive after chemotherapy [107] (Table 2).

There is evidence to suggest that lncRNAs are also involved in BL pathogenesis. For example, a study focused on MYC-regulated lncRNAs identified 13 differentially expressed lncRNAs. Among these, MYC-induced long non-coding RNA (MINCR) showed a positive correlation with MYC expression [116]. Long non-coding RNA, plasmacytoma variant translocation 1 (PVT1) has also been reported to be associated with *MYC* expression as well as with alterations in cell cycle-associated genes in the Raji cell line [117]. The antisense non-coding RNA in the INK4 locus (ANRIL) is another lncRNA that has been identified as a regulator of cell proliferation and apoptosis in multiple types of cancer, including BL, and does so by regulating TGF-β1 signaling [118]. LncRNA-ANRIL also promotes gastric cancer progression by enhancing NF-κB signaling [119] and regulates STAT3 in liver cancer [120]. Recently, Guo et al. identified a novel lncRNA (MCM3AP-AS1) that was upregulated in BL tissues and associated with poor prognosis [121]. Knockdown of MCM3AP-AS1 increased drug sensitivity, enhanced cell cycle progression, and facilitated apoptosis by regulating miR-15a/EIF4E and its downstream anti-apoptotic proteins in vitro and in vivo [121]. Another study highlighted the potential prognostic significance of lncRNA showing that the lncRNA deleted in lymphocytic leukemia 1 (*DLEU1*) may in part function as a tumor suppressor gene and was associated with treatment resistance in children and adolescents with BL in a miR-15a/16-1 dependent manner [122] (Table 2).

**Table 2:** Clinical potential of miRNAs/lncRNAs in BL.

miRNA/ncRNA	Putative Role	Clinical Potential	References
**hsa-miR-17**	OncomiR	Prognostic biomarker	[90]
**hsa-miR-21**	OncomiR	Prognostic biomarker	[94]
**hsa-miR-23a**	OncomiR	Prognostic biomarker	[123]
**has-miR-10a-5p**	OncomiR	Prognostic biomarker	[107]
***MCM3AP-AS1***	Oncogene	Prognostic biomarker	[121]
***DLEU1***	Tumor suppressor	Prognostic biomarker	[122]

## 4. Diffuse Large B-Cell Lymphoma

Diffuse large B-cell lymphoma (DLBCL) is the most common form of NHL in adults, accounting for more than 80% of cases of aggressive lymphoma [124]. DLBCL is a diverse group of B-cell lymphomas showing pathological and biological heterogeneity as well as differences in clinical presentation and outcome [125]. Gene expression profiling has identified three major molecular subtypes of DLBCL: germinal center B-cell-like (GCB) DLBCL, activated B-cell-like (ABC) DLBCL, and primary mediastinal large B-cell lymphoma (PMLBCL) [125,126]. GCB-DLBCL is characterized by frequent translocations involving the *BCL2* gene, *REL* amplifications, and somatic hypermutation of the immunoglobulin genes. Both ABC-DLBCL and PMBCL show constitutive activation of NF-κB signaling with the more aggressive ABC-DLBCL having a poorer clinical outcome [124,125,126,127]. Clinical responses to rituximab-CHOP (cyclophosphamide, doxorubicin, vincristine, prednisolone) immunochemotherapy, the current standard-of-care, are variable [128]. Thus, unfortunately, a large number of DLBCL patients will die of their disease. The precise mechanism underlying drug resistance in DLBCL has not yet been determined.

Recently, cellular miRNAs have contributed significantly to our understanding of the biology of DLBCL and have been incorporated into models for distinguishing different types of DLBCL, and as tools for differential diagnosis. Thus, a specific miRNAs signature including miRs-155, -221, -222, -146a, and -146b has been reported to distinguish GCB–DLBCL from ABC–DLBCL [89,129,130]. miR-155 expression is 10- to 30-fold higher in DLBCL cells than in normal circulating B cells and is significantly associated with treatment failure following R-CHOP [33,131].

Several miRNAs, including those containing *MYC*-regulated and NF-κB pathway associated miRNA (i.e., miRs-155, -29b, -146a, -17-3p, -365, -30b, -595, -663, -573, -26b, -374, -520d, -92, -let7f, -516-3p, -9, -629, -9*, -34b) can discriminate between BL and DLBCL or between DLBCL and follicular lymphoma (FL) (i.e., miR-330, -17-5P, -106A, -210) with an overall accuracy of around 98% [88,123,132,133].

Approximately 15% of DLBCL are EBV positive. The virus establishes a latency II or latency III pattern of viral gene expression with expression of all BART miRNAs except for BART15 and BART20. BART7, BART10, BART11-5p, BART16, and BART22 are the most abundantly expressed miRNAs [61,134]. EBV contributes to DLBCL by regulating key cell signaling pathways through its viral proteins and miRNAs. For instance, LMP1 is able to increase miR-34a, -146a, and -155 expression via the NF-κB pathway, while BART3, BART9, and BART17-5p can downregulate BCL6 and thereby increase NF-κB activation [135,136,137,138].

miRNA signatures are also associated with drug resistance and clinical outcome in DLBCL patients treated with immunochemotherapy (Table 3). For instance, miR-18a, -21, -181a, and -222 can predict OS and progression-free survival (PFS) in R-CHOP–treated DLBCL patients [129]. Upregulation of miR-455-3p and -33a was found to be associated with chemosensitivity, while upregulation of miR-125b, -130a, -224, -1236, and -520d-3p were associated with chemoresistance [129,139].

Numerous studies have suggested a close relationship between DLBCL and aberrant lncRNA expression [140,141,142,143,144,145] (Table 3). For instance, Zhu et al. showed a significantly different expression of lncRNAs in DLBCL cell lines compared to normal B cells; NAALADL2-AS2 exhibited the strongest upregulation, and NON-HSAT120161 the strongest downregulation [141]. Another study of 116 DLBCL patients confirmed the differential expression of lncRNAs between tumors and normal B cells wherein two-thirds of 2632 lncRNAs were not expressed in normal B cells and more than one-third of lncRNAs were differentially expressed between ABC and GCB subtypes [145]. Zhou et al. performed a comparative analysis of lncRNA profiles of a large cohort of DLBCL (*n* = 905) and identified a 17-lncRNA signature (ENTPD1-AS1, SACS-AS1, SH3BP5-AS1, RP11-101C11.1, AC009892.10, RP1-68D18.4, MIR600HG, RP11-278 J6.4, RP11-203B7.2, CSMD2-AS1, CTC-467 M3.1, RP4-788P17.1, RP11-553 L6.5, CRNDE, RP11-519G16.3, RP11-21 L19.1 and MME-AS1) able to classify GCB and ABC subtypes, and which also predicted OS and PFS [144]. In addition, a meta-analysis conducted by Sun et al. demonstrated that a 6-lncRNA signature (SACS-AS1, MME-AS1, CSMD2-AS1, RP11-360F5.1, RP1125K19.1, and CTC-467M3.1) could be used to classify patients with significantly different outcomes [146] (Table 3). Functionally, a number of lncRNAs have been shown to be involved in DLBCL pathogenesis. For example, SNHG16 can induce apoptosis of DLBCL cells in vitro [147]. SNHG14 could promote DLBCL immune evasion by regulating the PD-1/PD-L1 checkpoint [148]. Other lncRNA have been separately associated with different clinical features. For example, expression of HOTAIR, which can regulate PI3K/AKT/NF-kB signaling, is significantly correlated with tumor size, clinical stage, and poor prognosis [149]. NEAT1 is a marker of poor prognosis [150], while NONHSAG026900 is associated with a favorable prognosis [151]. MALAT1 is reported to be involved in chemotherapy sensitivity in DLBCL cell lines by enhancing autophagy-related proteins [152]; and PEG10, a lncRNA activated by MYC and reported to be upregulated in DLBCL tissues and in cell lines, acts as an oncomiR by promoting cell proliferation [153]. PEG10 expression also correlates with poor prognosis in DLBCL patients [154].

**Table 3:** Clinical potential of miRNAs/lncRNAs in DLBCL.

miRNA/ncRNA	Putative Role	Clinical Potential	References
**hsa-miR-155**	OncomiR	Prognostic biomarker	[155,156]
**hsa-miR-221/222**	OncomiR	Prognostic biomarker	[129]
**hsa-miR-146a**	OncomiR	Prognostic biomarker	[156]
**hsa-miR-146b**	OncomiR	Prognostic biomarker	[157]
**hsa-miR-18a**	OncomiR	Prognostic biomarker	[158]
**hsa-miR-21**	OncomiR	Prognostic biomarker	[159]
**hsa-miR-181a**	OncomiR	Prognostic biomarker	[129,160]
**hsa-miR-222**	OncomiR	Prognostic biomarker	[129]
***19-lncRNA***	Oncogene	Prognostic biomarker	[123]
***17-lncRNA***	Oncogene	Diagnostic and prognostic biomarker	[144]
***6-lncRNA***	Oncogene	Prognostic biomarker	[146]
***NAALADL2-AS2***	Oncogene	Diagnostic biomarker	[141]
***NON-HSAT120161***	Oncogene	Diagnostic biomarker	[141]
***PEG10***	Oncogene	Prognostic biomarker	[154]
***HOTAIR***	Oncogene	Prognostic biomarker	[149]
***NEAT1***	Oncogene	Prognostic biomarker	[150]
***NONHSAG026900***	Tumor suppressor	Prognostic biomarker	[151]

## 5. Nasopharyngeal Carcinoma

EBV is also an etiological agent in nasopharyngeal carcinoma (NPC) [161]. The EBV genome is detectable in almost all cases of undifferentiated NPC. EBV’s protein-coding genes, EBNA1 and LMP1, are crucially involved in disease pathogenesis [162,163]. The EBERs may also contribute to some of the characteristic features of NPC. For example, when introduced into a canine epithelial cell line (MDCK), EBER expression enhanced cell growth [164]. The EBERs were shown to induce an inflammatory response in NPC cells through Toll-like receptor 3 (TLR3) signaling [165]. Zhang et al. also reported that EBER-1 expression was negatively correlated with the intergenic non-coding RNA LINC00312, which may act as a tumor suppressor in NPC [166]. Other cellular lncRNAs are regulated by EBV in NPC. He at al. described the upregulation of three lncRNAs (MALAT1, AFAP1-AS1, AL359062) following EBV infection of immortalized normal nasopharyngeal epithelial (NP69) cells [167]. Although it is still unclear how EBV infection regulates signaling pathways affecting expression of these three lncRNAs, the recognition of crosstalk between EBV infection and lncRNAs could provide a novel mechanism for EBV-induced carcinogenesis.

There have also been efforts to elucidate the role of EBV-encoded miRNAs in NPC (Table 4). For instance, Chen et al. used deep sequencing technology to establish a complete BART miRNA transcriptome wherein 44 mature EBV microRNAs, including four novel subtypes, were identified from the 22 distinct precursor hairpins in the BART region [168]. Interestingly, a widespread sequence variation was detected in the 44 BART miRNAs [169,170]. Among the EBV BART miRNAs, BART3-3p, BART5-5p, and BART9-3p were highly upregulated [168]. In particular, BART5-5p was previously reported to regulate the pro-apoptotic gene *PUMA* and protect NPC cells from etoposide-induced apoptosis [171]. Meanwhile, BART7-3p was found to regulate epithelial-to-mesenchymal transition (EMT) which is one of the mechanisms implicated in NPC metastasis [172,173]. In a different study, BART1 was found to influence the expression of the oncogenic *PSAT1* and *PHGDH* genes in NPC [174], resulting in the activation of PTEN-dependent pathways, including PI3K/Akt, FAK-p130^Cas^, and Shc-MAPK/ERK1/2 signaling. With the activation of these signaling pathways, there is increased EMT favoring the migration, invasion, and metastasis of NPC [175].

BART7 contributes to the resistance of NPC cells to both cisplatin and radiation therapy [168]. In particular, BART7-3p was found to induce expression of *c-myc* and *c-jun* by activating the PTEN/PI3K/Akt pathway [176]. Additionally, BART8-3p promoted radioresistance in NPC by upregulating ATM/ATR signaling [177]. BART9 is also highly upregulated in EBV-positive NPC tumors and may play a role in repressing E-cadherin, thereby upregulating β-catenin to promote the metastatic spread of NPC [178]. In tumor tissues of a cohort of 106 NPC patients, BART10-3p expression was negatively correlated with *BTRC* expression and was found to be associated with poor prognosis [179]. BART10-3p promoted the invasion and migration of NPC cells by targeting BTRC and facilitating metastatic spread through EMT [175]. BART2-5p promoted the migration and invasion of EBV-negative NPC cells, whereas its genetic downregulation in EBV-positive NPC cells decreased metastasis [180]. BART2-5p also decreased expression of the RND3 Rho family GTPase to promote ROCK signaling, cell motility, and the metastatic behavior of NPC cells [180].

**Table 4:** EBV-encoded miRNAs in NPC.

miRNA/ncRNA	Putative Role	Clinical Potential	References
**BART5-5p**	OncomiR	Prognostic biomarker	[171]
**BART9-3p**	OncomiR	Prognostic biomarker	[172,173]
**BART1**	OncomiR	Prognostic biomarker	[175]
**BART8-3p**	OncomiR	Prognostic biomarker	[177]
**BART2-5P**	OncomiR	Prognostic biomarker	[180]
**BART8-3p**	OncomiR	Diagnostic biomarker	[181]
**BART10-3p**	OncomiR	Diagnostic biomarker	[182]
**BART19-3p**	OncomiR	Diagnostic biomarker	[183]

With potential clinical utility, there have been initiatives to detect circulating EBV-encoded miRNAs in serum, plasma, or whole blood of individuals with NPC. Using the Exiqon miRNA qPCR panel, Zou et al. identified a signature of five miRNAs (let-7b-5p, miR-140-3p, miR-192-5p, miR-223-3p, miR-24-3p) that were significantly upregulated in serum samples of patients with NPC compared with non-cancer controls [181]. In another study, Gao et al. performed qPCR on plasma from patients with NPC [182]. For recurrent NPC, plasma levels of BART2-3p, BART2-5p, BART5-3p, BART5-5p, BART6-3p, BART8-3p, BART9-5p, BART17-5p, BART19-3p, and BART20-3p were significantly upregulated. BART19-5p was the miRNA that performed best in identifying NPC in patients with undetectable levels of plasma EBV DNA. For recurrent NPC, BART8-3p and BART10-3p performed best in identifying cancer in EBV DNA undetectable plasma [182]. Wu et al. showed that BART19-3p was significantly upregulated in the serum and tumor tissues of NPC patients with a sensitivity and specificity of 71.7 and 72.3%, respectively, for the detection of cancer vs. non-cancer controls [183]. Furthermore, profiling miRNA expression levels in whole blood of patients with NPC led to the identification of two miRNA signatures (8-miRNA and 16-miRNA signatures) with high diagnostic accuracy for NPC. In particular, the 16-miRNA signature (hsa-miR-1224-3p, -1280, -155-5p, -1908, -1973, -296-5p, -361-3p, -425-5p, -4284, -4436b-5p, -4439, -4665-3p, -4706, -4740-3p, -5091, and -5091) is the first meaningful diagnostic signature to differentiate NPC from other head–neck tumors and healthy subjects [184].

## 6. Gastric Carcinoma

Up to 10% of gastric cancers (GC) are EBV-positive [185,186,187,188]. Indeed, a proposed classification separates GC into four subtypes: EBV-positive, microsatellite instable (MSI), chromosomal instable (CI), and genomically stable (GS). EBV-associated gastric cancer (EBVaGC) is usually localized in the proximal stomach (the cardia section and body) [189,190,191]. Adenocarcinomas of the gastroesophageal junction, but not esophageal adenocarcinomas, are also EBV-associated in some cases, suggesting that characteristics of the epithelial cells that are only found in the gastroesophageal junction and the proximal stomach are important for persistent EBV infection [192]. EBVaGC is likely to be ‘immunogenic’ given the observation of high levels of tumor infiltrating lymphocytes (TILs) which are a positive prognostic marker [193,194]. Unlike many other solid tumors, PD-L1 expression in EBVaGC is predominantly observed in the immune infiltrate and can predict success with immune checkpoint inhibitors [195,196]. EBVaGC expresses EBNA1, EBER1/2, and BART miRNA. LMP2A expression is present in around one-half of all cases [186,188,197,198]. EBNA1 contributes to the downregulation of both p53 and transforming growth factor-beta (TGF-β) signaling, and promotes tumor growth by enhancing NF-κB activation in both EBVaGC and NPC [199,200,201]. The EBERs are also implicated in the pathogenesis of EBVaGC, for example, by increasing the expression of insulin-like growth factor 1, which can act as an autocrine growth factor [202,203,204].

The effects of these EBV genes may also be indirect as they can affect the expression of other miRNAs that contribute to cancer proliferation [188]. For instance, a decrease in pri-miR-200 transcription was observed in gastric carcinoma cell lines infected with EBV, resulting in a decrease of E-cadherin expression and loss of cell adhesion [205].

BART14-3p is highly expressed in EBVaGC [206]. In contrast, BART22 expression, while high in other tumors expressing a type I form of EBV latency, is low in EBVaGC [206]. EBV miRNAs were shown to target multiple points in the apoptotic cascade in EBVaGC. For example, BART4-5p was found to reduce the activity of the pro-apoptotic protein Bid leading to reduced apoptosis in gastric cancer cell lines [206]. BART5-3p also downregulates p53 expression in NPC and EBVaGC [207]. Multiple BART miRNAs, including BART1, BART3, BART9, BART11, and BART12, can reduce expression of the pro-apoptotic gene, Bcl-2-interacting mediator of cell death (*BIM*) in GC cells [208]. BART20-5p inhibited the apoptosis of gastric carcinoma cells through its ability to target the Bad protein [209]. BART10-3p and BART22 are also implicated in worse 5-year OS and are associated with lymph node metastasis and activation of the Wnt signaling pathway in EBVaGC [210].

Differential expression of cellular miRNAs between malignant and benign gastric mucosa was also observed. Although hsa-miR-21 and -155 are involved in inflammation, which is a feature of both benign and malignant tissues, both miRNAs are upregulated in gastric cancer tissues [211]. Other upregulated miRNAs in gastric carcinomas include hsa-miR-196a, -196b, -185, and -let-7i. Downregulated cellular miRNAs include hsa-miR-18a, 34a, 187, -200a, -423-3p, -484-, and -744 [211].

Several lncRNAs are also associated with EBVaGC pathogenesis. For example, SNHG8 was found to be more highly expressed in EBVaGC than in EBV-negative gastric carcinomas. Although the exact mechanism is still unclear, SNHG8 was found to interact with EBV proteins LF3, BHLF1, BHRF1, and BNLF2a which regulate the expression of cellular genes *TRIM28*, *EIF4A2*, *NAP1L1*, *PLD3*, *RPL18A*, and *TRPM7* that play a role in the pathogenesis of gastric cancer [212]. The clinical potential of measuring ncRNA in gastric cancer is summarized in Table 5.

**Table 5:** miRNAs/lncRNAs in gastric carcinoma.

miRNA/ncRNA	Putative Role	Clinical Potential	References
**BART4-5p**	OncomiR	Diagnostic biomarker	[206]
**BART5-5p**	OncomiR	Diagnostic biomarker	[213]
**BART20-5p**	OncomiR	Prognostic biomarker	[214]
**BART10-3p**	OncomiR	Prognostic biomarker	[210]
**BART22**	OncomiR	Prognostic biomarker	[210]
**hsa-miR-196a**	OncomiR	Diagnostic biomarker	[211]
**hsa-miR-196b**	OncomiR	Diagnostic biomarker	[211]
**hsa-miR-185**	OncomiR	Diagnostic biomarker	[211]
**hsa-miR-let-7i**	OncomiR	Diagnostic biomarker	[211]
***SNHG8***	Oncogene	Diagnostic biomarker	[212]

## 7. Role of ncRNA in Immune Evasion

We briefly outline below the emerging importance of ncRNA in immune evasion in EBV-associated malignancies.

Recent evidence supports a role for EBV miRNAs in modulating host cytokine signaling during different stages of the viral life cycle. For example, in an analysis of TCGA data, expression of the BART cluster of EBV-encoded miRNAs (BART2, BART4, BART5, BART18, BART22) was shown to be associated with expression of IL-10 and TGF-β1, inhibitory cytokines that can suppress host immune responses to EBV, and with PD-1/PD-L1 expression and poor survival [215]. Some miRNAs (e.g., BART6-3p) can inhibit RIG-I signaling and the type I interferon response, both crucial for innate antiviral immunity [216]. Another key mediator of the innate immune response is the NLRP3 inflammasome. NLRP3 is a protein complex activated by pathogen-associated molecular patterns (PAMPs). Exosome-mediated secretion of BART15 can abrogate the inflammatory capacity of NLRP3 [217]. In nasal NK cell lymphoma, BART20-5p and BART8 suppress IFN-γ-STAT1 signaling [218]. Targets of EBV miRNAs were also shown to be enriched for genes with functions in innate and adaptive immune responses [219]. Moreover, the increased levels of the EBV miRNA correlated with reduced immune cell infiltration in both BL and GC [219]. It is noteworthy that this same study showed that EBV miRNAs form thermodynamically stable complexes with their targets at a higher efficacy than cellular miRNAs, indicating that they are likely to override any regulation of immune targets by cellular miRNA [219].

BART miRNAs can induce a regulatory phenotype in tumor-associated macrophages (TAMs) by enhancing their expression of IL-10, TNF-α, and arginase 1 [70]. As described earlier, BART13-3p is highly expressed in cHL and can be delivered to TAMs via exosomes promoting TAM differentiation towards a pro-tumor phenotype. Similarly, BART1, BART2, and BHRF1-2 can suppress secretion of IL-12 from infected B cells thus inhibiting MHC class II-dependent neo-antigen processing, the differentiation of CD4+ Th1 cells, and the recognition and elimination by CD4+ effector T cells [220]. Moreover, miRNA-mediated downregulation of the proinflammatory cytokine IL-12 can abrogate CD8+ cytotoxic T cell surveillance of EBV-infected cells [221].

miRNA can induce expression of immune checkpoint molecules. Elevated expression of immune checkpoint molecules, including PD-L1, is a feature of several EBV-associated malignancies (e.g., cHL, NPC, GC) [195,222,223,224,225]. Increased PD-L1 expression could be mediated by the exosomal delivery of EBV miRNAs from transformed cells to cells of the tumor microenvironment. Mapping of EBV miRNA sequences from the Cancer Genome Atlas (TCGA) has shown that the BART cluster is significantly associated with PD-1/PD-L1 expression and aggressive phenotypes in EBV-associated malignancies [215]. BART5-5p can induce PIAS3 downregulation, STAT3 activation and, subsequently, PD-L1 upregulation [213]. However, it is likely that many of the underlying mechanisms governing how EBV-encoded miRNAs regulate immune checkpoints are yet to be fully discovered. EBV miRNAs exert their immune regulatory functions in a combinatorial fashion with their host cellular counterparts. Therefore, the ‘immune targetome’ of EBV miRNAs may only be fully appreciated with a systems medicine approach to characterize the full network of virus-host interactions.

## 8. Clinical Potential of miRNAs and Other Non-Coding RNAs

**Figure 3:**
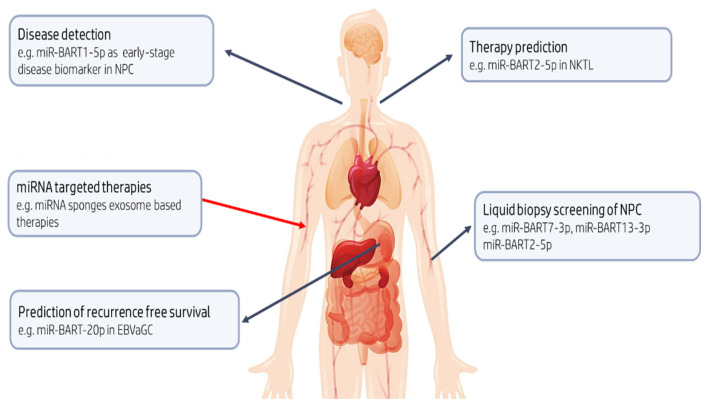
Potential clinical utility of EBV-encoded miRNAs. BART13-3p could aid in the detection of preclinical NPC, a cancer, which often presents late and when advanced, is associated with poor prognosis. In NKTL, which is characterized by early invasion and metastasis, miR-BART2-5p may have a role as diagnostic and predictive biomarker with the potential to identify those likely to respond to therapy. Identification of the risk of recurrence after potentially curative resection in patients with gastric cancer remains a priority; BART20-5p levels in EBVaGC might be useful in predicting recurrence free survival. Finally, rapidly emerging mRNA therapeutic technologies can be adopted to inhibit or mimic the action of miRNAs with oncogenic or tumor suppressive roles, respectively.

As outlined below, EBV miRNAs and other ncRNAs may have clinical utility in the diagnosis of cancer, in more accurate outcome prediction, or in therapy (Figure 3).

Serological assays to detect levels of antibodies to EBV antigens, e.g., EBV viral capsid antigen (VCA) and EBV early antigen (EA), have been useful as an aid to diagnosis and in disease monitoring in some settings. Thus in NPC, combined antibody panels are able to improve diagnostic utility in endemic regions [226]. However, an unmet need exists for novel biomarkers for early screening in at risk populations given that EBV serology tests often have low positive predictive value [215]. The assessment of circulating cell-free EBV-DNA load is also a promising minimally invasive tumor marker [227]. However, there remains no consensus on its clinical use. In NPC, for example, this is mainly because cutoff levels and timing of EBV DNA detection differ between studies [228,229].

Circulating miRNAs can be actively and selectively secreted from living cells and passively leaked from apoptotic cells [230]. As such, circulating microRNA detection in ‘liquid biopsy’ has potential as a clinical biomarker [42]. For instance, the measurement of EBV-encoded miRNAs in combination with other biomarkers may be an optimal strategy for early NPC detection [231]. Among the BART miRNAs that have been analyzed and validated in clinical samples, BART7-3p, BART13-3p, and BART 2-5p appear to be the best performing NPC-selective biomarkers [230,231,232,233,234,235,236]. Separate studies on Chinese and Malaysian populations showed that plasma BART7-3p levels were higher in NPC patients, especially in advanced stages, compared to clinically healthy controls [234,235,236]. However, since these EBV-encoded miRNAs have also been detected in some non-NPC samples, appropriate ‘normal ranges’ may need to be defined if these biomarkers are to be adopted in the clinical setting. The diagnostic accuracy of BART13-3p detection outperformed existing methods, i.e., EBV DNA load and EBV IgA titers in some studies [235]. BART 2-5p was also proposed as a sensitive and specific biomarker for screening preclinical NPC patients in a high-risk population [233]. Circulating BART2-5p, BART7-3p, BART13-3p, and BART1-5p levels could also be used to identify patients with nasal natural killer/T-cell lymphoma (NKTL) [230]. It should be noted, however, that the presence of miRNAs in tissue samples does not guarantee their concordant presence in plasma. The reasons for such discrepancies are not known, but have implications for the development of clinical assays [211].

While the aforementioned studies made use of serum or plasma samples, another promising application is the less invasive use of nasopharyngeal brush sampling to detect and quantify elevated levels of BART1-5p in NPC. In addition to over 90% sensitivity, specificity, and a positive correlation between high BART1-5p and tumor progression, this approach can also detect early-stage NPC missed through conventional methods [237].

miRNAs might also be used to provide more accurate information on likely patient outcomes. For example, BART7 overexpression contributes to resistance to cisplatin chemotherapy and radiotherapy in NPC [238]. Similarly, high BART20-5p expression may predict recurrence-free survival for patients with EBVaGC [214]. Large-scale studies will be required to confirm the utility of BART7-3p and BART13-3p levels in monitoring treatment efficacy as they have been observed to be significantly reduced post-therapy [231,234], as well as BART7-3p levels as a potential prognostic marker for distant metastasis in NPC [234]. Moreover, additional studies on the use of BART17-5p to monitor relapse or presence of residual cells in NPC patients are needed [232].

There is also the potential to use miRNAs in therapeutic applications. Highly overexpressed EBV BART-miRNAs in epithelial tumors have classically been the target. For example, miRNA-sponges or anti-miRNA oligonucleotide therapeutic ‘antagomirs’ have been utilized to effectively suppress EBV-driven tumors [176,239]. A new technology is ‘exosome-based therapy’ with loaded immunostimulatory cargo as cell-free immunotherapy for EBV-associated malignancies [240]. The tumor-suppressive role for other miRNAs (miR-216b) through inhibition of KRAS-related AKT and ERK signaling has also been shown in NPC as a proof of concept [241].

The recently described EBV circular RNAs (circRNAs) have expanded the spectrum of potential therapeutic viral targets [242,243,244,245,246]. Moreover, the better stability of circRNAs compared to their linear counterparts, makes them attractive biomarker candidates in liquid biopsy. One such possibility is EBV-circLMP2A, which has been shown to induce and maintain stemness in EBVaGC; high expression of EBV-circLMP2A was also significantly correlated with metastasis and poor prognosis [247]. In NPC, EBV-circRPMS1 promotes epithelial-mesenchymal transition (EMT) which could serve as an early marker of metastasis [248]. Finally, long non-coding RNAs of EBV (BART lncRNAs and BHLF1 lncRNA) are increasingly recognized to be of importance in different facets of the viral life cycle, including virus replication [249].

## 9. Concluding Remarks

The emerging field of ncRNA biology is already providing multiple opportunities, not only to better understand the pathogenesis of malignant disease, but also in the development of new biomarkers of disease, in defining novel therapeutic targets and/or even as alternative therapies. Although EBV was the first human virus identified to express miRNA, challenges remain before applications involving EBV miRNA, or other ncRNA encoded by the virus, can be routinely adopted into clinical practice.
